# A meta-analysis of changes in gut microbiota structure following bariatric surgery

**DOI:** 10.1097/JS9.0000000000002977

**Published:** 2025-07-18

**Authors:** Min Chen, Tai-Chun Tang, Yao-Yao Chen, Hui Zheng

**Affiliations:** a Department of Colorectal Diseases/Clinical Medicine, Hospital of Chengdu University of Traditional Chinese Medicine, Chengdu, China; b The Third Hospital/Acupuncture and Tuina School, Chengdu University of Traditional Chinese Medicine, Chengdu City, China

**Keywords:** bariatric surgery, gut microbiota structure, meta-analysis, systematic review

## Abstract

**Background:**

Bariatric surgery is a common intervention for obesity, yet its impact on gut microbiota remains unclear.

**Objective:**

This systematic review and meta-analysis evaluated changes in gut microbiota composition before and after bariatric surgery.

**Methods:**

We searched PubMed, Embase, Web of Science, and Cochrane Library up to October 2024 for randomized controlled trials (RCTs) and observational studies reporting pre- and post-surgery gut microbiota composition. Two reviewers independently screened studies, extracted data, and assessed bias using the Cochrane risk of bias tool and the Newcastle–Ottawa Scale (NOS). Primary outcomes included alpha diversity changes (Chao and Shannon indices), while secondary outcomes focused on relative abundance changes at phylum, family, and genus levels. Data were pooled using random-effects models.

**Results:**

Among 3670 screened articles, 45 were included, with 30 achieving NOS scores ≥7 and one trial having some concerns in the risk of bias assessment. Post-surgery, alpha diversity significantly increased but with high heterogeneity (Chao index: SMD 0.50, 95% CI 0.01–0.99, *P* = 0.046, I^2^ = 87.3%; Shannon index: SMD 0.37, 95% CI 0.04–0.70, *P* = 0.028, I^2^ = 90.2%). Meta-regression identified age and geographic region as heterogeneity sources. Both RYGB and LSG surgery increased the abundance of *Akkermansia, Bacteroides, Streptococcus*, and *Veillonella*, but the abundance of *Bifidobacterium* and *Lactobacillus* was reduced after LSG surgery.

**Conclusion:**

Bariatric surgery significantly increases gut microbiota alpha diversity, with notable genus-level changes that indicate probiotic supplementation may be beneficial post-LSG. Owing to the high heterogeneity in taxonomic findings, further studies are needed to robustly establish the causal effects of specific surgical procedures on individual taxa.

## Introduction

Bariatric surgery has become a cornerstone in the management of severe obesity, particularly for individuals who fail to achieve sustained weight loss through non-surgical interventions. Procedures such as Roux-en-Y gastric bypass (RYGB), sleeve gastrectomy (SG), and adjustable gastric banding (LAGB) have demonstrated significant efficacy in weight reduction and improvement of obesity-related diseases, including type 2 diabetes, hypertension, and obstructive sleep apnea^[1,2]^. A network meta-analysis found that RYGB and sleeve gastrectomy SG were associated with the highest excess weight loss rates, with SG achieving 81.2% excess weight loss at 1 year^[3]^. Additionally, bariatric surgery has shown remarkable remission rates for type 2 diabetes, with RYGB achieving remission in 98% of cases^[4]^. Current guidelines recommend bariatric surgery for individuals with a body mass index (BMI) ≥40 or ≥35 accompanying with diabetes or other related diseases. Emerging evidence also supports its use in individuals with obesity with a BMI of 30–35 and poorly controlled type 2 diabetes^[5,6]^.

A growing body of evidence suggests that bariatric surgery exerts a profound influence on gut microbiota composition. Numerous studies have demonstrated that bariatric surgical procedures, particularly RYGB and sleeve gastrectomy SG, induce substantial alterations in the gut microbial profile. These modifications are characterized by an increase in beneficial bacterial species, such as *Akkermansia muciniphila*, and a reduction in *Firmicutes*, which are typically associated with obesity^[7,8]^. Nevertheless, inconsistencies persist across various studies, particularly regarding the Firmicutes/Bacteroidetes ratio, a biomarker frequently associated with metabolic health. For instance, while some studies reported significant changes in the Firmicutes/Bacteroidetes ratio post-surgery^[9–11]^, others have observed no significant alterations in this ratio^[12,13]^.

Based on the aforementioned literature, three key questions remain to be addressed. First, does the alpha-diversity of gut microbiota increase following bariatric surgeries? Second, are there differences in the composition of gut microbiota – at the phylum, family, and gene levels – among different surgical procedures, particularly between the two most commonly performed techniques, RYGB and SG^[14,15]^? This aspect has been rarely explored in previous systematic reviews and meta-analyses. Third, what are the main confounding factors or sources of heterogeneity that influence the changes in gut microbiota after bariatric surgery?

Given the evidence presented above, we aim to conduct a systematic review and meta-analysis to elucidate the alterations in gut microbiota following bariatric surgery in individuals with obesity and to assess whether these changes are influenced by the specific type of surgical procedure performed.

## Methods

This study was prospectively registered with the International Prospective Register of Systematic Reviews (PROSPERO) under the registration number CRD42024588946, accessible via https://www.crd.york.ac.uk/prospero/. The research was conducted and reported in strict adherence to the PRISMA (Preferred Reporting Items for Systematic Reviews and Meta-Analyses)^[16]^ and the AMSTAR (Assessing the Methodological Quality of Systematic Reviews) guidelines^[17]^, ensuring methodological rigor and transparent reporting throughout the systematic review process. This study was conducted without using artificial intelligence (AI) tools in accordance with the TITAN Guidelines 2025^[18]^.

### Search strategy

A comprehensive and systematically designed search strategy was developed to identify studies investigating alterations in the gut microbiome following bariatric surgery. The detailed search syntax and Boolean operators are presented in Supplemental Digital Content, Table S1, available at: http://links.lww.com/JS9/E727. We conducted an exhaustive literature search across multiple electronic databases, including PubMed, Web of Science, and Embase, encompassing all publications up to 10 October 2024 (the final search date). The search strategy incorporated key terms such as “Bariatric Surgery,” “gut,” and “microbiome” in title, abstract, and full-text fields. To ensure maximal retrieval of relevant studies, we supplemented the electronic search with manual screening of reference lists from identified reviews and related publications. The inclusion criteria were limited to primary human studies, with no restrictions applied to publication language or geographical origin.

### Study screening

Two reviewers independently screened the retrieved studies for inclusion. In the first stage, two independent reviewers evaluated the titles and abstracts against the predefined eligibility criteria. Studies that clearly did not meet the inclusion criteria were excluded. Any discrepancies between reviewers were resolved through discussion or consultation with a third reviewer. In the second stage, the full texts of potentially relevant studies were retrieved and assessed independently by the same two reviewers. Studies that met the inclusion criteria were selected for data extraction, while those that did not were excluded with reasons documented. The screening process was reported in accordance with the PRISMA guideline, and a PRISMA flow diagram was generated to illustrate the study selection process, including the number of records identified, excluded, and included at each stage.

**HIGHLIGHTS**

- Differential mechanisms of surgical types on microbiota: This study is the first to systematically reveal distinct impacts of Roux-en-Y gastric bypass (RYGB) and laparoscopic sleeve gastrectomy (LSG) on the gut microbiota. *Akkermansia* significantly increases only in the RYGB group, and the reduction of Firmicutes is also observed exclusively in the RYGB group, suggesting that RYGB may have greater advantages in improving metabolic parameters. These findings fill the gaps in the existing literature regarding the effects of different surgical procedures on the microbiota.
- Clinical significance of key microbial genera: The study clarifies the central roles of genera such as *Bifidobacterium, Streptococcus*, and *Veillonella* in post-surgical metabolism and immune regulation. The decrease in *Bifidobacterium* may be associated with post-surgical changes in the nutritional environment, while the co-metabolic pathway of *Streptococcus* and *Veillonella* is crucial for energy expenditure. These results provide a theoretical basis for optimizing post-surgical metabolic health through probiotic supplementation or targeted interventions in the future.
- In-depth analysis of heterogeneity sources: The research indicates that, in addition to surgical procedures, factors such as regional differences, age, follow-up duration, diet, and antibiotic use all influence the microbiota changes after bariatric surgery. This conclusion emphasizes the necessity of controlling multiple confounding factors in future studies. It is recommended to adopt standardized methods, such as uniform dietary records and long-term follow-up, to reduce heterogeneity and enhance the comparability of research results.

The inclusion criteria comprised: (1) study designs including randomized controlled trials (RCTs), prospective or retrospective cohort studies, and case-control studies; (2) adult participants (aged ≥18 years) who underwent bariatric surgeries such as Roux-en-Y gastric bypass (RYGB), sleeve gastrectomy (SG, including vertical SG (VSG) and laparoscopic SG [LSG]), and adjustable gastric banding (AGB); (3) studies reporting gut microbiome composition data post-surgery; and (4) no language restrictions. Excluded were case reports, editorials, conference abstracts, and non-peer-reviewed works; studies lacking quantitative data or only reporting qualitative outcomes; studies with insufficient data for analysis even after contacting authors; and redundant studies or secondary analyses without new original data. This framework balances inclusivity with rigor, focusing on relevant research designs, surgical populations, and outcomes while excluding non-quantitative or duplicative works.

### Data extraction

Data extraction was conducted systematically using a standardized form developed and piloted on a subset of included studies to ensure accuracy and consistency. Two independent reviewers extracted data, with discrepancies resolved through discussion or consultation with a third reviewer. Key extracted information included study characteristics (author, year, country, design, sample size, follow-up duration), population details [age, sex, BMI, surgery type, total weight loss (%)], outcome measures (alpha diversity metrics, relative abundance of bacterial taxa), and statistical analysis methods (*P* values and effect sizes). Missing or unclear data were requested from study authors when possible.

### Outcome definition

The primary outcome was the change in alpha diversity of the gut microbiome before and after bariatric surgery. Alpha diversity, which reflects the richness and evenness of microbial species within a sample, was assessed using established metrics, including the Shannon index (combining species richness and evenness), Chao index (estimating total species richness), Simpson index (emphasizing species richness and evenness), and OTU (operational taxonomic unit). These metrics provide a comprehensive evaluation of microbial diversity within individuals.

Shannon and Chao indices were the primary measures used. The Shannon index captures both species richness and evenness, making it sensitive to subtle shifts in microbial community structure after bariatric surgery. The Chao index estimates total species richness, especially when rare taxa are undersampled. It complements Shannon by focusing on taxon count rather than abundance distribution. When specific metrics were not reported, “richness” and “evenness” were used as general terms in the analysis.

The secondary outcomes focused on changes in relative abundance of specific microbial taxa at the phylum, family, and genus levels. Relative abundance was calculated as the proportion of sequences assigned to a particular taxon relative to the total sequences in the sample, typically derived from 16S rRNA gene sequencing data. This approach allows for the identification of shifts in the composition of the gut microbiome following surgery. Both outcomes were evaluated at predefined time points pre- and post-surgery to assess temporal changes in microbial ecology.

### Quality assessment

The methodological quality of the included studies with observational design was assessed using the Newcastle–Ottawa Scale (NOS), a validated tool for evaluating the quality of non-randomized studies in meta-analyses. The NOS assesses studies based on three domains: selection of study groups, comparability of groups, and outcome assessment. Each domain includes specific criteria, with a maximum score of 9 points indicating the highest quality. Two independent reviewers performed the quality assessment, and any discrepancies were resolved through discussion or consultation with a third reviewer. Studies were categorized as high quality (score ≥7), moderate quality (score 5–6), or low quality (score ≤4) based on their total NOS scores.

The methodological quality of randomized controlled trials was assessed using the Cochrane risk of bias 2.0 tool. The tool focuses on five key domains – randomization process, deviations from protocol, completeness of outcome data, selective reporting, and other sources of bias – to determine if bias is low risk, high risk, or if there are some concerns.

The results of the quality assessment were used to interpret the robustness of the findings and to explore potential sources of bias in the included studies.

### Statistical analysis

Data synthesis was conducted to address two primary research questions: (1) What are the overall changes in alpha diversity following bariatric surgery? and (2) What are the changes in relative abundance of gut microbiota post-surgery?

For the analysis of alpha diversity, the overall changes after surgery were assessed by calculating the standardized mean difference (SMD) between pre- and post-surgery measurements, along with the corresponding 95% confidence interval (95% CI). The selection of alpha diversity metrics was prioritized according to the following hierarchy: Chao index, Shannon index, Simpson index, operational taxonomic units (OTUs), richness, and evenness. The SMDs were pooled using both fixed-effect (common-effect) and random-effect models. Heterogeneity was evaluated using the I^2^ statistic, with I^2^ ≤ 40% indicating negligible heterogeneity, in which case the fixed-effect model results were adopted; otherwise, the random-effect model results were utilized. A similar meta-analytical approach was applied to assess changes in gene-level relative abundance.

To investigate potential sources of heterogeneity, meta-regression analyses were performed for key variables, including age, the percentage of participants with diabetes, follow-up duration, geographic region, baseline BMI, and total weight loss (%). Subgroup analyses were conducted when a specific variable significantly contributed to heterogeneity, as indicated by a *P* <0.05 in the meta-regression model.

To examine the association between surgical types and changes in gut microbiota, heatmaps were generated to illustrate changes in taxonomic levels at the phylum, family, and gene levels, as well as their correlations with surgical types. Surgical procedures were categorized as Roux-en-Y gastric bypass (RYGB), sleeve gastrectomy (SLG), mixed types of RYGB and SLG, and other surgical types. Only taxonomic changes reported by at least two original studies included in the systematic review were considered significant.

All statistical analyses were performed using R software (version 4.4.1, available at www.r-project.org).

## Results

### Literature search and results

**Figure 1.**
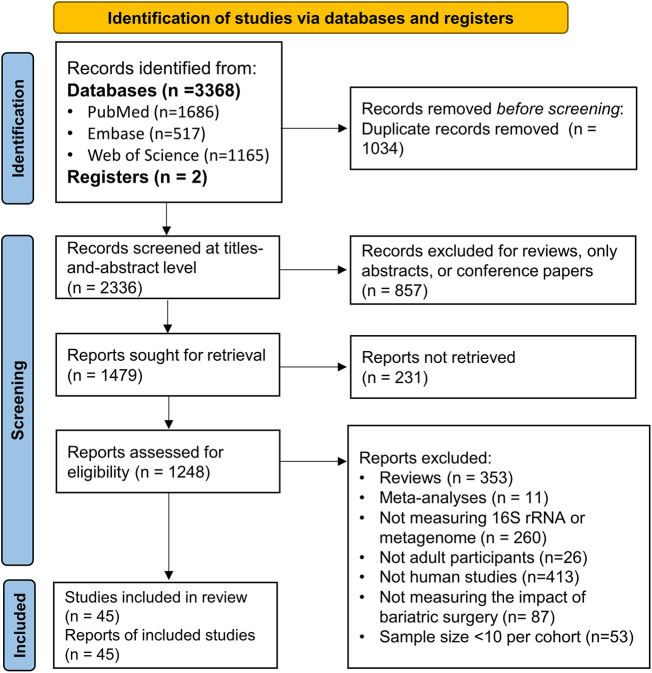
Flowchart of the study.

The systematic literature search yielded 3,370 potentially relevant records. Following rigorous screening procedures, 45 studies met the inclusion criteria and were included in the final analysis^[10,13,19–62]^. The study selection process is illustrated in Figure 1. The sample sizes across studies ranged from 10 to 92 participants. Among the included studies, adult participants with median or mean ages ranging from 30 to 53 years were enrolled. Diabetes prevalence was reported in 33 studies, with a median proportion of 30% among participants.

The most frequently reported surgical procedures were Roux-en-Y gastric bypass (RYGB) and laparoscopic sleeve gastrectomy (LSG), with 24 studies reporting RYGB and 21 studies reporting LSG. The majority of studies conducted follow-up assessments at standardized time points: 3 months (8 studies), 6 months (16 studies), and 12 months (15 studies) postoperatively. Geographically, the studies were distributed as follows: 6 studies (13.3%) from the Americas, 16 studies (35.6%) from Asia, and 18 studies (40%) from Europe. The reported total weight loss ranged from 11% to 54%, with a median value of 30%. Comprehensive characteristics of the included studies are presented in Table 1. Rationale of the exclusion of studies conducted in recent 5 years was listed in Supplemental Digital Content, Table S2, available at: http://links.lww.com/JS9/E728.

**Table 1:** Characteristics of included studies

Trial	Country	N*	Age (yr, SD/range)	N, T2Da	Intervention	Follow-up period	Regions	BMI (kg/m^2^)b	Total weight loss (%)b	Design
Morán-Ramos *et al*, 2024	Mexico	20(8)	38.8(8.6)	6	RYGB	12 months	America	Pre-surgery, 45.7(35.2-77.6); Post-surgery, 32.9(24.4-46.8)	53.6	Prospective observational study
Assal *et al*, 2020	Brazil, France	25(25)	45.8(8)	25	RYGB	12 months	America	Pre-surgery: 46.4(5.5), Post-surgery: 32.7(3.5)	29.4	Prospective observational study
Dong *et al*, 2023	USA	18(18)	37.1(9.4)	NA	LSG	12 months	America	44.7(4.9)	24.3	Prospective observational study
Prudêncio *et al*, 2023	Brazil	20(20)	47(6.5)	20	RYGB	3 months	America	Pre-surgery, 46.5(5.9)	33.7	Prospective observational study
Akagbosu *et al*, 2024	USA	12(8)	15(10–18)	8	VSG	5(3-7) months	America	Pre-surgery, 48.7; Post-surgery, 39.9	17.8	Prospective observational study
Wu *et al*, 2024	USA, Canada	23(19)	48(12)	7	LSG	6 months	America	Pre-surgery, 42.3(5.3)	24	Prospective observational study
Dang *et al*, 2022	Canada, USA	80(52)	47.7 (8.7)–47.9(9.7)–47(9.9)	6	RYGB, SG, non-operative controls	9 months	America	Pre-surgery: SG, 40.8(5.7); RYGB, 42.9(4.2)	RYGB, 26.1; SG, 20.1	Prospective observational study
Lin *et al*, 2019	USA, Taiwan	20(NA)	36.2(9.9)	0	CWLT, SG	3 months	America, Asia	Pre-surgery: CWLT, 36(4.6), SG, 35.9(4)	DC, 3.3; SG, 18.5	Prospective observational study
Shen *et al*, 2019	USA, Spain	26(NA)	NA	8	GB, SG	12 months	America, Europe	46.1(6.3)	33.5	Prospective observational study
Fouladi *et al*, 2021	USA, Brazil, UK, Denmark	61(26)	43.5 ±10.0–45.1 ± 6.1	NA	RYGB	12 months	America, Europe	Pre-surgery 44.7(7.1)	NA	Prospective observational study
Fukuda *et al*, 2022	Japan	10(4)	45	4	LSG	12 months	Asia	Post-surgery 43.9	30.3	Prospective observational study
Wei *et al*, 2022	China	20(9)	(33 ± 11) years	11	RYGB	12 months	Asia	Simple obesity: pre-surgery 55(19), post-surgery 40(14); Obesity with diabetes: pre-surgery 39(8), post-surgery 29(6)	Simple obesity, 27.3; Obesity with diabetes, 25.6	Retrospective observational study
Tabasi *et al*, 2021	Iran	126(96)	37.3(6.3)	16	LSG	12 months	Asia	Pre-surgery 43.3(5.4); Post-surgery 29.5(4.4)	32	Prospective observational study
Han *et al*, 2022	Korea	52(NA)	NA	NA	RYGB, SG	12 months	Asia	>35	N/A	Prospective observational study
Huang *et al*, 2022	Taiwan	13(5)	42.4(8.6)	13	LSG	2 years	Asia	Pre-surgery 35.6(3.2); Post-surgery 26.0(3.1)	27.3	Prospective observational study
Kural *et al*, 2022	Turkey, China	27(NA)	18–65	NA	LSG	3 months	Asia	>40 or >35 with comorbidities	28	Prospective observational study
Chen *et al*, 2023	China	40(15)	30.3(6.7)	NA	LSG	3 months	Asia	Pre-surgery 42.7(4.2); Post-surgery 31.3(5.6)	22.1	Prospective observational study
Shi *et al*, 2021	China	14(NA)	NA	0	RYGB	6 months	Asia	NA	NA	Prospective observational study
Kaniel *et al*, 2022	Israel	32(16)	44.5(12.3)	3	OAGB	6 months	Asia	41.7(6.6)	67.8	Prospective observational study
Özdemir *et al*, 2023	Turkey	34(27)	45.0(8.5)	4	SG	6 months	Asia	48.8(40.5–56.3)	24.7	Case control study
Kim *et al*, 2022	Korea	58(34)	18–60	14	SG, RYGB	6 months	Asia	Pre-surgery 39.2(30.1-62.1); Post-surgery 30.6(22.3-45.6)	21.2	Prospective observational study
Chen, *et al*, 2017	China	24(10)	51.5(9.6)	24	RYGB	6 months	Asia	Pre-surgery: 46.3(4.7); Post-surgery: 38.1(3.1)	17.7	Prospective observational study
Kikuchi *et al*, 2018	Japan	44(22)	40.7 ±2.0–48.0 2.5–41.0 ± 5.1 years	25	LSG, LAGB	6 months	Asia	>30 kg/m^2^	LSG, 51.8; LAGB, 31.9	Prospective observational study
Golzarand *et al*, 2023	Iran	42(42)	NA	NA	LRYGB, LSG	6 months	Asia	NA	NA	Prospective observational study
Chen *et al*,2020	China	87(60)	30.9(9.2)	33	LSG, RYGB	9.6 months	Asia	Pre-surgery: 40.8(10.7)	29.4	Prospective observational study
Zhang *et al*, 2024	China	15	NA	NA	SLG	NA	Asia	NA	NA	Prospective observational study
Paganelli *et al*, 2019	Netherlands	45(36)	44(9.29)	4	RYGB, SG	12 months	Europe	Pre-surgery: RYGB: 43(4.13), SG: 42.9(6.56); Post-surgery: RYGB: 31.52(3.86), SG: 30.81(5.35)	RYGB: 26.7; SG: 28.2	Prospective observational study
Palleja *et al*, 2016	Denmark, China	13(8)	>20	7	RYGB	12 months	Europe	>40 kg/m^2^ or >35 kg/m^2^ with diabetes or hypertension	11	Prospective observational study
Aron-Wisnewsky *et al*, 2019	France	61(61)	36.9(9.9)	9	AGB or RYGB	12 months	Europe	Pre-surgery: RYGB, 46.3(6.2), AGB, 43(2.2); Post-surgery: RYGB, 32.2(7), AGB, 35.7(2.3)	RYGB, 30.7; AGB, 17.1	Prospective observational study
Lau *et al*, 2021	Portugal, France	20(7)	53–58	20	RYGB, SMT	12 months	Europe	Pre-surgery: surgical group 33.6, medical group 32; Post-surgery: surgical group 24.6, medical group 30.5	Surgical group 25.5, medical group 4.9	Randomized controlled trial
Wijdeveld *et al*, 2023	Netherlands	67(51)	47.9(8.9)	NA	RYGB	12 months	Europe	Pre-surgery, 39.9(4.1)	28	Prospective observational study
Pérez-Rubio *et al*, 2023	Spain	12(8)	52.5(2.4)	NA	RYGB	12 months	Europe	Pre-surgery, 45.8(0.3)	15.4	Prospective observational study
Salazar *et al*, 2022	Spain	40(22)	NA	12	SG, RYGB	3 months	Europe	RYGB, pre-surgery 57.2, post-surgery 39.9; SG, pre-surgery 47.3, post-surgery 33.8	RYGB, 50.1; SG, 49	Prospective observational study
Campisciano *et al*, 2018	Italy	20(NA)	18–65	NA	LGB, LSG	3 months	Europe	> 40 kg/m2or 35-40 kg/m2 with obesity-related comorbidities	NA	Prospective observational study
Sanchez-Alcoholado *et al*, 2019	Spain	28(20)	NA	NA	RYGB, SG	3 months	Europe	Pre-surgery: RYGB: 43.7(5.3), SG: 46.9(6.6); Post-surgery: RYGB: 37.2(4.4); SG: 38.4(5.8)	RYGB, 17.8; SG, 18.5	Prospective observational study
Sanchez-Carrillo *et al*, 2021	Spain	40(NA)	NA	NA	RYGB, LSG	3 months	Europe	Pre-surgery 46; Post-surgery 36.8	20	Prospective observational study
Bernard *et al*, 2023	France	32(32)	38.4(1.3)	0	VSG	6 months	Europe	43.1(0.7)	19.3	Prospective observational study
Farup *et al*, 2021	Norway	92(88)	43.6(8.5)	4	CWLT, RYGB	6 months	Europe	Pre-surgery: 41.9(3.5), Post-surgery: 30.6(3.7)	27	Matched case control design and prospective cohort design
Patrone *et al*, 2016	Italy	11(NA)	48.3(range 35–64)	6	BIB	6 months	Europe	Pre-surgery: 47.46 (7.5); Post-surgery: 40.7(5.9)	16.6	Prospective observational study
Kong *et al*, 2013	France	30(30)	NA	7	RYGB	6 months	Europe	NA	NA	Prospective observational study
Palmisano *et al*, 2020	Italy	50(40)	44.5(range 20-62)	8	LSG, RYGB	6 months	Europe	Pre-surgery: 44.5(5.5)	27.3	Prospective observational study
Tedjo *et al*, 2023	Netherlands	51(NA)	47.5(9.4)	14	SG	6 months	Europe	Pre-surgery, 23(1.5) for healthy group, 38.7(3.6) for obese group; Post-surgery, 31.1 (6.6) for obese group	25.1	Prospective observational study
Gutiérrez-Repiso *et al*, 2021	Spain	61(34)	MedDiet: 64.0 ± 4.7 years, BS: 47.5 ± 5.5 years, VLCKD: 42.6 ± 10.8years	NA	MedDiet, VLCKD, SG	6 months	Europe	Pre-surgery: surgical group 45(5), MedDiet group 33.4(3.3), VLCKD 33.0(1.4); Post-surgery: surgical group 37.3(4.3), MedDiet 30.6(3.3), VLCKD 28.5(1.3)	SG, 18.6; MedDiet 8.9; VLCKD, 13.9	Prospective observational study
Prykhodko *et al*, 2024	Sweden	15(11)	41 (19–61)	3	RYGB	Up to 6 years	Europe	Pre-surgery, 37.6(31-44)	23.3	Prospective observational study
Li *et al*, 2021	UK, USA	107(76)	43.1	NA	RYGB, LSG	2 years	Europe, America	Pre-surgery 47.5	NA	Prospective observational study
Ahmad *et al*, 2023	Australia	17(11)	51.5(7.8)	4	SG	6 months	Oceania	41.4(9.7)	30.1	Prospective observational study

AGB: adjustable gastric banding; BIB: bilio-intestinal bypass; CWLT: conservative weight loss therapy; DCET: dietary counseling and exercise training; GB: gastric bypass; LAGB: laparoscopic adjustable gastric banding; LSG: laparoscopic sleeve gastrectomy; MedDiet: Mediterranean diet; NA: not applicable; OAGB: one anastomosis gastric bypass; RYGB: Roux-en-Y gastric bypass; SG: sleeve gastrectomy; SLG: sleeve-like gastrectomy; SMT: standard medical therapy; VLCKD: very-low-calorie ketogenic diet; VSG: vertical sleeve gastrectomy.

* Total number of participants and number of female cases.

^a^ Number of participants with type 2 diabetes.

^b^ Post-surgical BMI and changes in body weight were evaluated based on the latest observation during the follow-up period.

Quality assessment using the NOS revealed that 16 studies (36.3%) scored 6 points, 22 studies (50%) scored 7 points, 4 studies (9%) achieved 8 points, and 2 studies (4.7%) attained the highest score of 9 points. One randomized controlled trial was assessed as having some concerns in the risk of bias evaluation. Detailed NOS assessment scores are provided in Supplemental Digital Content, Table S3 (available at: http://links.lww.com/JS9/E729), and detailed risk of bias assessments are presented in Supplemental Digital Content, Table S4 (available at: http://links.lww.com/JS9/E730).

### Changes in alpha-diversity following bariatric surgery

A significant increase in alpha-diversity after bariatric surgery was observed across multiple indices, including the Chao index (random effects model: SMD 0.46, 95% CI 0.31 to 0.61, *P* < 0.001, I^2^= 87.3%), Shannon index (random effects model: SMD 0.37, 95% CI 0.04 to 0.70, *P* = 0.028, I^2^ = 90.2%), and other assessed variables (Table 2; Supplemental Digital Content, Figures S1–S6, available at: http://links.lww.com/JS9/E726). Notably, substantial heterogeneity was observed in all meta-analyses evaluating changes in alpha-diversity, with I^2^ values exceeding 60% (Table 2).

**Table 2:** Changes in alpha diversity after surgery

Measures	Model	No. of studies	SMD (95%CI)	*Z* value	*P* value	I^2^ value
Chao index	Fixed effect model	22	0.46 (0.31 to 0.61)	6.1	<0.001	87.30%
	Random effect model	22	0.5 (0.01 to 0.99)	1.99	0.046	
Evenness index	Fixed effect model	78	0.349 (0.138 to 0.559)	3.2174	<0.001	74.805.1%
	Random effect model	78	0.4351 (0.0412 to 0.829)	2.1458	0.0321	
OTUs	Fixed effect model	17	0.45 (0.31 to 0.6)	6.05	<0.001	91.60%
	Random effect model	17	0.92 (−0.04 to 1.87)	1.87	0.061	
Richness	Fixed effect model	11	1.48 (1.22 to 1.74)	11.29	<0.001	91.80%
	Random effect model	11	1.7 (0.68 to 2.71)	3.28	0.001	
Shannon index	Fixed effect model	43	0.18 (0.09 to 0.27)	3.91	<0.001	90.2%
	Random effect model	43	0.37 (0.04 to 0.7)	2.2	0.028	
Simpson index	Fixed effect model	23	−0.5619 (−1.090.63 to −0.030.26)	−2.080.81	0.038416	87.56.40%
	Random effect model	23	−0.9136 (−2.61.8 to 0.781.07)	−1.060.50	0.2962	

OTUs: operational taxonomic units; SMD: standardized mean difference; 95%CI: 95% confidence interval.

Chao index: a measure of species richness, estimating the total number of species in a community. Evenness index: a measure of how evenly individuals are distributed among species in a community. Richness: the number of different species present in a community. Shannon index: a measure of diversity that considers both species richness and evenness. Simpson index: a measure of diversity that emphasizes the dominance of the most abundant species. Fixed effect model: a statistical model that assumes all studies share a common effect size; Random effect model: a statistical model that accounts for variability between studies, allowing for different effect sizes.

To explore potential sources of heterogeneity, meta-regression analyses were conducted to assess the influence of six baseline variables – age, percentage of type 2 diabetes, follow-up duration, study region, BMI, and total weight loss after surgery – on four alpha-diversity parameters with at least 10 studies. Region emerged as a significant contributor to heterogeneity in OTUs and richness, with age also identified as a contributing factor (Table 3). Subgroup analyses stratified by region revealed that the Asian population exhibited the most pronounced increase in alpha-diversity (random effects model: SMD 2.52, 95% CI −1.75 to 6.79), compared to the American population (random effects model: SMD 0.52, 95% CI −0.4 to 1.44) and the European population (random effects model: SMD −0.12, 95% CI −0.82 to 0.58) (Supplemental Digital Content, Figures S7, available at: http://links.lww.com/JS9/E726). Subgroup analysis by age was not performed due to missing age data in at least 50% of the included studies.

**Table 3:** Meta-regression analysis for Chao and Shannon index

Moderators	Estimate (95%CI)	*Z* value	*P* value
Chao index			
Age	−0.13 (−0.29 to 0.04)	−1.534	0.125
Type 2 diabetes	−0.22 (−2.94 to 2.5)	−0.158	0.875
Follow-up time	0.04 (−0.04 to 0.12)	1.03	0.303
Asian region	2.26 (0.18 to 4.34)	2.13	0.033
BMI	0.06 (−0.07 to 0.2)	0.896	0.37
Weight loss percent	0.03 (−0.03 to 0.09)	1.01	0.314
Shannon			
Age	−0.002 (−0.05 to 0.04)	−0.069	0.945
Type 2 diabetes	0.8 (−0.6 to 2.19)	1.119	0.263
Follow-up time	0.002 (−0.06 to 0.06)	0.05	0.956
Asian region	0.86 (−0.16 to 1.89)	1.651	0.099
BMI	0.04 (−0.06 to 0.14)	0.794	0.428
Weight loss percent	−0.02 (−0.04 to 0.01)	−1.16	0.248
OTUs			
Age	0.04 (0.01 to 0.07)	2.886	0.004
Type 2 diabetes	0.38 (−3.53 to 4.29)	0.19	0.85
Follow-up time	0.0002 (−0.126 to 0.127)	0.003	0.998
Asian region	−0.27 (−1.63 to 1.08)	−0.397	0.692
BMI	0.05 (−0.08 to 0.17)	0.778	0.437
Weight loss percent	−0.003 (−0.03 to 0.02)	−0.233	0.815
Richness			
Age	−0.24 (−0.31 to −0.18)	−7.433	<0.001
Type 2 diabetes	−3.46 (−6.92 to 0)	−1.96	0.05
Follow-up time	−0.017 (−0.7 to 0.66)	−0.048	0.962
European region	1.58 (−2.31 to 5.46)	0.796	0.426
BMI	0.13 (−0.13 to 0.4)	0.987	0.324
Weight loss percent	−0.004 (−0.22 to 0.22)	−0.233	0.971

BMI: body mass index; 95%CI: 95% confidence interval.

Chao index: a measure of species richness, estimating the total number of species in a community. Shannon index: a measure of diversity that considers both species richness and evenness. OTUs: operational taxonomic units, used to classify groups of closely related organisms based on genetic similarity. Richness: the number of different species present in a community.

### Changes in relative abundance at the gene level following bariatric surgery

Table 4 summarizes the changes in relative abundance at the gene level after bariatric surgery. A significant decrease in the relative abundance of *Bifidobacterium* was observed (random effects model: SMD −0.92, 95% CI −1.74 to −0.09, *P* = 0.03, *I*^2^= 96.8%). In contrast, significant increases were noted for *Oscillospira* (random effects model: SMD 1.24, 95% CI 0.67 to 1.81, *P* < 0.001, *I*^2^= 38%), *Streptococcus* (random effects model: SMD 1.85, 95% CI 0.57 to 3.14, *P* = 0.005, *I*^2^= 97.6%), and *Veillonella* (random effects model: SMD 1.64, 95% CI 0.3 to 2.97, *P* = 0.016, *I*^2^= 97.5%). Forest plots illustrating the meta-analysis results for relative abundance at the gene level are provided in Supplemental Digital Content, Figures S8–S14, available at: http://links.lww.com/JS9/E726.

**Table 4:** Gene level meta-analysis

Genes	Model	No. of studies	SMD (95%CI)	*Z* value	*P* value	I^2^
Bifidobacterium	Fixed effect model	17	−0.31 (−0.46 to −0.16)	−4.08	<0.001	96.80%
	Random effect model	17	−0.92 (−1.74 to −0.09)	−2.17	0.03	
Enterobacteriaceae	Fixed effect model	8	0.88 (0.66 to 1.09)	7.88	<0.001	94.20%
	Random effect model	8	0.45 (−0.64 to 1.54)	0.81	0.418	
Lactobacillus	Fixed effect model	7	−0.22 (−0.47 to 0.04)	−1.66	0.097	95.90%
	Random effect model	7	−0.5 (−1.89 to 0.88)	−0.71	0.476	
Oscillospira	Fixed effect model	2	1.21 (0.77 to 1.65)	5.38	<0.001	38%
	Random effect model	2	1.24 (0.67 to 1.81)	4.25	<0.001	
Prevotella	Fixed effect model	11	−0.06 (−0.22 to 0.09)	−0.78	0.438	86.60%
	Random effect model	11	0.09 (−0.56 to 0.74)	0.27	0.789	
Streptococcus	Fixed effect model	14	1.31 (1.13 to 1.48)	14.34	<0.001	97.60%
	Random effect model	14	1.85 (0.57 to 3.14)	2.82	0.005	
Veillonella	Fixed effect model	11	1.26 (1.08 to 1.45)	13.17	<0.001	97.50%
	Random effect model	11	1.64 (0.3 to 2.97)	2.4	0.016	

SMD: standardized mean difference; 95% CI: 95% confidence interval.

No. of studies: number of studies included in the analysis. I^2^ value: a measure of heterogeneity among studies, indicating the percentage of variation due to differences rather than chance. Fixed effect model: a statistical model that assumes all studies share a common effect size. Random effect model: a statistical model that accounts for variability between studies, allowing for different effect sizes.

### Changes in taxonomic composition at the phylum level associated with surgical types

**Figure 2.**
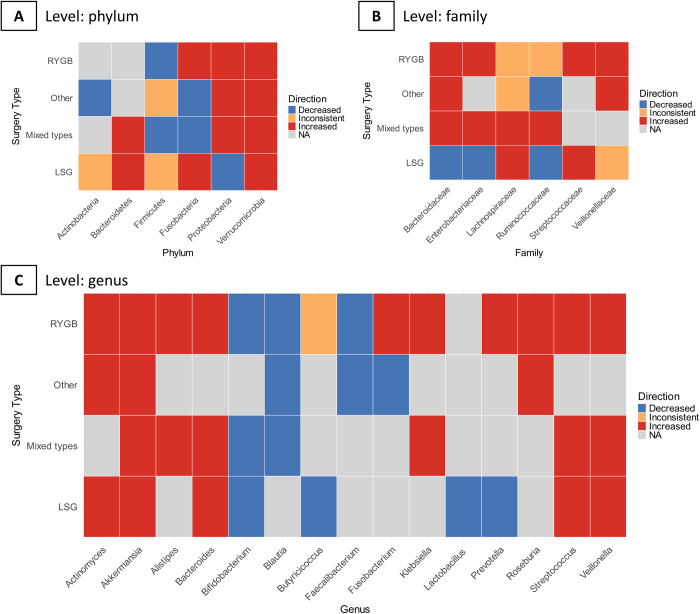
Changes in gut microbiota composition. Abbreviation: RYGB: Roux-en-Y gastric bypass; SLG: sleeve-like gastrectomy. The figure presents a heatmap illustrating the changes in gut microbiota composition across multiple taxonomic levels, including phylum, family, and genus. The vertical axis represents different surgical procedures, while the horizontal axis denotes the taxonomic levels. Changes at each taxonomic level are supported by findings from at least two included studies. The reported microbial alterations are categorized based on their directional trends, including “Increased,” “Decreased,” “Inconsistent,” and “NA” (not applicable or not reported). This visualization provides a comprehensive overview of the microbial shifts associated with various surgical interventions, highlighting consistent and divergent patterns across studies.

Figure 2A illustrates the changes in taxonomic composition at the phylum level associated with the types of bariatric surgeries. Roux-en-Y gastric bypass (RYGB) was associated with an increase in *Fusobacteria, Proteobacteria*, and *Verrucomicrobia*, alongside a decrease in *Firmicutes*. In contrast, laparoscopic sleeve gastrectomy (LSG) was associated with an increase in *Bacteroidetes, Fusobacteria*, and *Verrucomicrobia*, but a decrease in *Proteobacteria*.

### Changes in taxonomic composition at the family level associated with surgical types

Figure 2B presents the changes in taxonomic composition at the family level associated with surgical types. RYGB was linked to an increase in *Bacteroidaceae, Enterobacteriaceae, Streptococcaceae*, and *Veillonellaceae*. LSG, on the other hand, was associated with an increase in *Lachnospiraceae* and *Streptococcaceae*, but a decrease in *Bacteroidaceae, Enterobacteriaceae*, and *Ruminococcaceae*.

### Changes in taxonomic composition at the gene level associated with surgical types

Figure 2C demonstrates the changes in taxonomic composition at the gene level associated with surgical types. RYGB was associated with an increase in *Actinomyces, Akkermansia, Alistipes, Bacteroides, Fusobacterium, Klebsiella, Prevotella, Roseburia, Streptococcus*, and *Veillonella*. LSG was linked to an increase in *Actinomyces, Akkermansia, Bacteroides, Streptococcus*, and *Veillonella*, but a decrease in *Bifidobacterium, Butyricicoccus, Lactobacillus*, and *Prevotella*.

## Discussion

Our systematic review and meta-analysis yielded three key findings. First, a significant increase in alpha diversity was observed following bariatric surgery, as demonstrated by the Chao index (SMD 0.50, 95% CI 0.01 to 0.99, *P*= 0.046) and the Shannon index (SMD 0.37, 95% CI 0.04 to 0.70, *P* = 0.028), alongside other metrics such as OTUs, richness, and evenness. Meta-regression analysis identified age and geographic region as significant contributors to heterogeneity across studies. Second, at the genus level, significant decreases in *Bifidobacterium* (SMD −0.92, 95% CI −1.74 to −0.09, *P* < 0.001) and increases in *Oscillospira* (SMD 1.24, 95% CI 0.67 to 1.81, *P* < 0.001), *Streptococcus* (SMD 1.85, 95% CI 0.57 to 3.14, *P* = 0.005), and *Veillonella* (SMD 1.64, 95% CI 0.30 to 2.97, *P* = 0.016) were observed. Third, distinct patterns of gut microbiota alterations were associated with different surgical procedures. Increases in *Actinomyces, Akkermansia, Bacteroides, Streptococcus*, and *Veillonella* were commonly observed in both RYGB and LSG, but the abundance of *Bifidobacterium* and *Lactobacillus* was reduced after LSG surgery.

### Comparison of previous meta-analysis

Our study found significant increases in alpha-diversity and distinct shifts in microbial composition. The observed increase in alpha-diversity aligns with the hypothesis that bariatric surgery restores microbial richness, which is often reduced in obesity^[63,64]^. This finding was consistent with previous systematic reviews^[65–67]^.

The impact of differential surgeries on the composition of gut microbiota was not consistent among our meta-analysis and previous studies. For instance, while previous studies emphasize the role of Akkermansia in metabolic improvements^[68,69]^, our study and one previous systematic review^[67]^ found a significant increase in Akkermansia, but not the other two systematic reviews^[65,66]^. In addition, we also found that Akkermansia only increased in the RYGB group, and it had no significant changes in the LSG group; the finding was not reported in previous systematic reviews. According to this finding, we speculated that RYGB might have better performance than LSG in improving metabolic parameters. Similarly, the reduction in Firmicutes, often linked to weight loss in the literature^[70,71]^, was significant only in the RYGB group in our study. Our systematic review also found that *Bifidobacterium* and *Lactobacillus* abundance decreased following LSG but not RYGB. This phenomenon could be explained by the fact that LSG primarily affects the microbiota through gastric volume reduction and decreased ghrelin secretion, thereby reducing genera such as *Bifidobacterium* and *Lactobacillus*. In contrast, RYGB differs from LSG due to its gastrointestinal diversion and malabsorption mechanisms. These findings suggest that probiotic supplementation post-LSG may be beneficial.

The heterogeneity observed in our meta-analysis, particularly in alpha-diversity measures, underscores the complexity of microbiota responses to bariatric surgery. Meta-regression analysis identified region and age as potential contributors to this heterogeneity, highlighting the need for future studies to account for these variables. Additionally, the varying anatomical and nutritional impacts of RYGB and LSG may explain the differential effects on microbial composition^[63]^. For instance, RYGB, which involves rerouting the gastrointestinal tract, may create a more pronounced shift in microbial ecology compared to LSG, which primarily reduces stomach volume. Furthermore, the duration of follow-up may influence the stability and observed diversity of post-surgical microbiota, as microbial communities may take months to stabilize after surgery^[66,72]^.

Bariatric surgery induces profound alterations in the gut microbiome, with *Bifidobacterium, Streptococcus*, and *Veillonella* emerging as key players in post-surgical metabolic and immune outcomes. *Bifidobacterium* contributes to enhanced gut barrier integrity, reduced systemic inflammation, and improved insulin sensitivity through the production of short-chain fatty acids (SCFAs), such as acetate and butyrate, and by modulating appetite via glucagon-like peptide-1 (GLP-1) signaling^[73]^. *Streptococcus* exhibits a dual role in post-surgical health: beneficial species like *Streptococcus gordonii* support metabolic homeostasis through lactate production, which *Veillonella* subsequently metabolizes into propionate, an SCFA that regulates energy expenditure and mitigates fat accumulation^[74]^. Conversely, pathogenic *Streptococcus* species may exacerbate inflammation and insulin resistance, underscoring the importance of post-surgical microbial surveillance^[75]^. *Veillonella* also serves as a critical node in microbial networks, enhancing exercise performance by metabolizing lactate; however, its overgrowth in dysbiotic states may predispose individuals to systemic infections^[76]^. Future research should prioritize elucidating the mechanistic roles of these genera in post-surgical outcomes, particularly their contributions to SCFA production, immune modulation, and microbial cross-talk. Clinical trials investigating probiotic supplementation with *Bifidobacterium* and targeted interventions modulating the *Streptococcus-Veillonella* axis could provide innovative strategies for optimizing metabolic health and preventing dysbiosis in individuals receiving bariatric surgery. Additionally, longitudinal monitoring and personalized microbiome profiling may refine post-surgical care, enabling the development of tailored therapeutic approaches to improve outcomes.

Several microbial shifts observed post-bariatric surgery may contribute directly or indirectly to metabolic improvements. For instance, the increased abundance of *Oscillospira* has been associated with improved insulin sensitivity and reduced inflammation, potentially through modulation of intestinal barrier function and bile acid metabolism^[77]^. Similarly, the rise in *Veillonella* and *Streptococcus* may enhance lactate-to-propionate conversion, a pathway linked to increased energy expenditure and reduced adiposity. Furthermore, reductions in *Bifidobacterium*, although seemingly counterintuitive, may reflect adaptive changes in response to altered nutrient availability and gut physiology following surgery^[78]^. These microbial alterations likely interact with host metabolism via multiple pathways, including short-chain fatty acid production, bile acid transformation, and regulation of incretin hormone secretion.

This study has several limitations that should be acknowledged. First, the included studies exhibited significant heterogeneity in terms of demographics, surgical techniques, and methodologies for microbiota analysis, which may have influenced the pooled results. Although meta-regression identified age and geographic region as sources of heterogeneity, residual confounding factors, such as dietary habits and antibiotic use, were not fully accounted for. Inconsistent follow-up time between the articles is also a potential source of heterogeneity. For instance, pre- and post-operative dietary patterns can significantly shape gut microbial composition. Variations in calorie intake, macronutrient distribution (e.g., fat vs. fiber intake), and adherence to post-surgical dietary guidelines across studies may have contributed to discrepancies in microbial changes observed after surgery. Similarly, differential use of antibiotics – particularly perioperative prophylactic regimens or long-term prescriptions – could alter microbial communities independently of surgical anatomy, potentially masking or exaggerating the true effects of bariatric procedures on the microbiome.

Second, the majority of included studies were observational, limiting the ability to infer causality between bariatric surgery and microbiota changes. Third, the reliance on relative abundance data rather than absolute quantification of microbial taxa may obscure true changes in microbial load. Additionally, the lack of long-term follow-up data in many studies restricts our understanding of the durability of these microbiota alterations. Finally, the exclusion of non-English studies and potential publication bias may have impacted the generalizability of the findings. Future research should address these limitations by incorporating standardized methodologies, longer follow-up periods, and mechanistic studies to better understand the clinical implications of these microbiota changes.

Bariatric surgery significantly increases gut microbiota alpha diversity and induces consistent shifts in key microbial genera, including reduced *Bifidobacterium* and elevated *Oscillospira, Streptococcus*, and *Veillonella*. These findings highlight the profound impact of surgery on gut microbial ecology. The reduction in beneficial taxa following laparoscopic sleeve gastrectomy (LSG) suggests that probiotic supplementation may be beneficial. However, high heterogeneity and limited mechanistic insights necessitate further longitudinal and functional studies to establish causality and explore the clinical relevance of these microbial changes in post-surgical metabolic outcomes.

## Data Availability

The data used in this systematic review and meta-analysis are publicly available.

## Research registration unique identifying number (UIN)

PROSPERO (CRD42024588946).
